# Use of the Iron-Responsive *RBT5* Promoter for Regulated Expression in Candida albicans

**DOI:** 10.1128/msphere.00305-22

**Published:** 2022-07-18

**Authors:** Yinhe Mao, Norma V. Solis, Anupam Sharma, Max V. Cravener, Scott G. Filler, Aaron P. Mitchell

**Affiliations:** a Department of Microbiology, University of Georgia, Athens, Georgia, USA; b Lundquist Institute for Biomedical Innovation at Harbor-UCLA Medical Center, Torrance, California, USA; University of Texas Health Science Center

**Keywords:** *Candida albicans*, gene expression, genetics, hyphal development

## Abstract

Engineered conditional gene expression is used in appraisal of gene function and pathway relationships. For pathogens like the fungus Candida albicans, conditional expression systems are most useful if they are active in the infection environment and if they can be utilized in multiple clinical isolates. Here, we describe such a system. It employs the *RBT5* promoter and can be implemented with a few PCRs. We validated the system with *RBT5* promoter fusions to two genes that promote filamentation and polarized growth, *UME6* and *HGC1*, and with *efg1*Δ/Δ mutants, which are defective in an activator of filamentous growth. An *RBT5* promoter fusion to either gene enabled filamentous growth of an *efg1*Δ/Δ mutant of strain SC5314 in iron-limited media, including RPMI with serum and yeast extract-peptone-dextrose with bathophenanthrolinedisulfonic acid. The *RBT5-UME6* fusion promoted filamentation of *efg1*Δ/Δ mutants in RPMI with serum of four other clinical C. albicans isolates as well. In a mouse model of disseminated candidiasis, the *RBT5-UME6* fusion promoted filamentation of the SC5314 *efg1*Δ/Δ mutant in kidney tissue, an indication that the *RBT5* promoter is active in the iron-limited host environment. The *RBT5* promoter expands the conditional expression toolkit for C. albicans genetics.

**IMPORTANCE** Genetic strategies have been vital for mechanistic analysis of biological processes. Here, we describe a genetic tool for the fungal pathogen Candida albicans.

## INTRODUCTION

Analysis of gene function often tests the impact of a gene alteration on phenotype. Such alterations can include reductions or increases in gene function or expression (1–3), as well as sequence variations that can result in quantitative or qualitative functional changes (4, 5). For pathogens like Candida albicans, the focus of our study, it is important that the impact of genetic alteration is manifested during proliferation in infection models. This feature allows assessment of the potential role of a gene in virulence-associated processes.

C. albicans is commensal on mucosal surfaces of the gastrointestinal and urogenital tracts in healthy individuals and can cause severe systemic infections in at-risk patients (6). Our understanding of virulence determinants is based mainly on deletion mutations, which cause a loss of gene function (7). However, as discussed in depth by Rai et al. (3), gene overexpression approaches have exceptional value for analysis of genetic redundancy, functional sufficiency, and epistasis or pathway relationships. Gene overexpression studies have been vital for study of virulence-associated processes that include the yeast-hypha transition and biofilm development (3).

Several constitutive promoters, such as P*_ACT1_* and P*_TDH3_*, are employed in C. albicans to induce stable high-level expression of the downstream gene (8, 9). Several conditional promoters regulate the expression of genes in a specific condition. For instance, the expression of genes under the control of P*_PCK1_*, P*_MAL2_*, or P*_MET3_* can be shut off by medium with glucose or methionine and cysteine (10–12). For conditional expression that can be controlled *in vitro* or *in vivo*, the P*_TET-_*_OFF_ and P*_TET-_*_ON_ systems respond to tetracycline analogs that do not affect metabolism (13). Thus, many platforms are already available for C. albicans gene overexpression studies.

Does the C. albicans research community need yet another gene overexpression platform? The answer is an unequivocal “maybe.” We sought features in our system that may be relevant to other investigators’ interests. We wanted the ability to overexpress a gene in a murine infection model; we wanted a system that worked with multiple clinical C. albicans isolates; we wanted a system that used a native C. albicans promoter to avoid the spiral of materials requests, material transfer agreement negotiations, and shipping and handling costs that can try an investigator’s patience and pocketbook. We expect that the *RBT5* promoter will be a useful arrow in the *Candida* geneticist’s quiver.

## RESULTS AND DISCUSSION

### Design and construction of an *RBT5*-based gene expression system.

**FIG 1:**
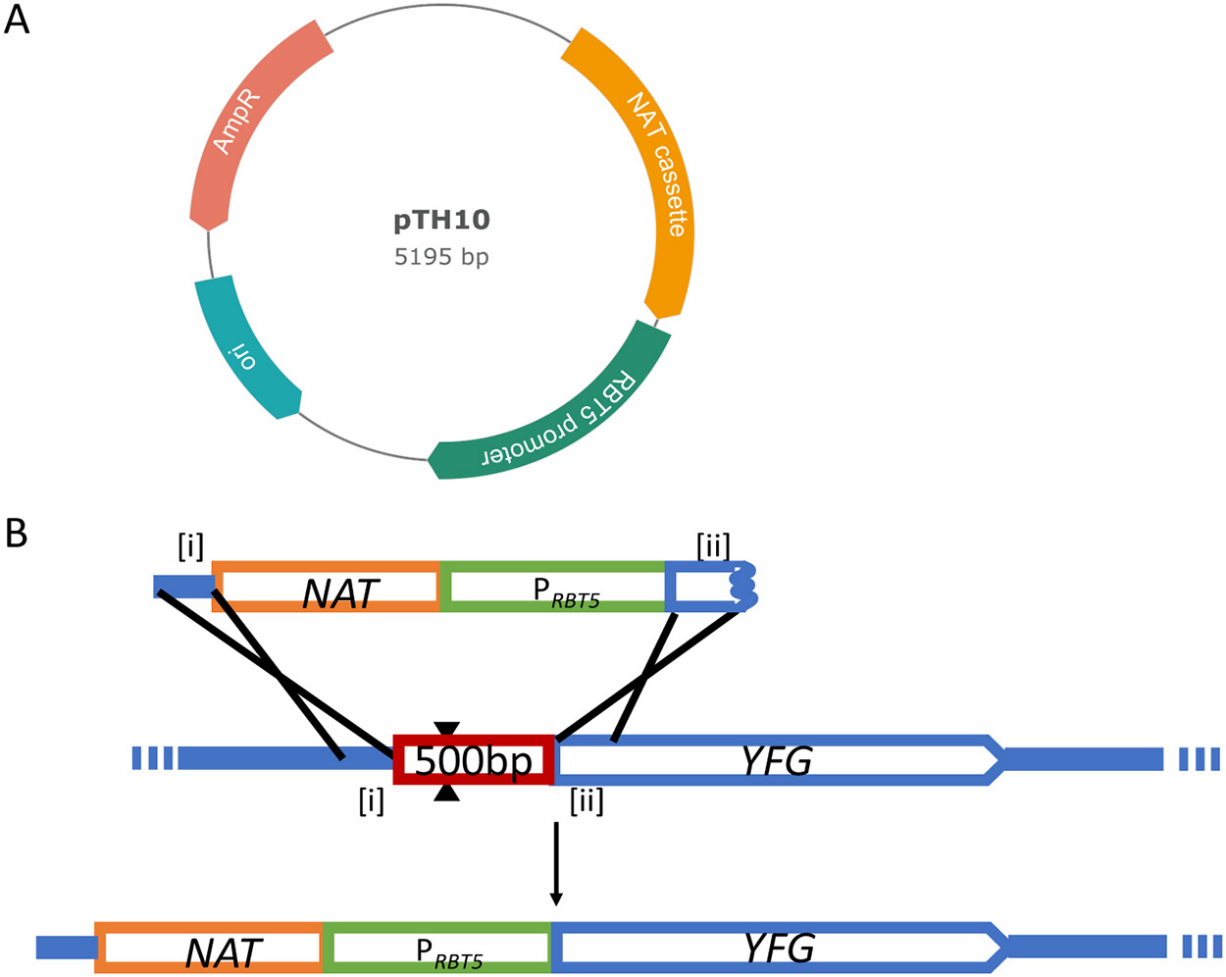
P*_RBT5_* expression system. (A) DNA segments containing the *NAT1* marker and *RBT5* promoter, assembled as PCR products in plasmid pTH10. (B) Sequence [i], with homology to the upstream region of *YFG*, is appended to the cassette with a long primer containing an 80-bp region of homology. Sequence [ii], with homology to the start of the *YFG* open reading frame, is also appended to the cassette with long primer containing an 80-bp region of homology. The cassette is transformed into a recipient strain along with DNA cassettes expressing *CAS9* and a single guide RNA targeting upstream of the *YFG* locus. The double-strand break introduced by Cas9 complexed with an sgRNA targeting the *YFG* upstream region is indicated by two black triangles. Expected homologous recombination events are depicted as single crosses, and together they should yield a locus containing Nat^R^ and P*_RBT5_* fused to the *YFG* open reading frame.

To achieve regulated overexpression of target genes in C. albicans, we designed a cassette containing the ~1-kbp *RBT5* 5′ region and the nourseothricin resistance (*NAT*) marker for selection of transformants (Fig. 1A). *RBT5* was chosen because its RNA displays a large expression difference between iron-replete and iron-limited growth conditions (14) and it is highly expressed *in vivo* (15). In addition, its modest size facilitates DNA manipulations. The *NAT* expression cassette, from plasmid pCJN542 (16), has been used for transformation of several different clinical isolates (17). These two components were assembled in plasmid pTH10 (Fig. 1B), and the *RBT5* segment was sequence verified.

### *RBT5* promoter activity *in vitro*.

We first tested the system with the *UME6* gene in the SC5314 strain background. Ume6 activates filamentation, so expression of *UME6* can be inferred from cell morphology (18). We used PCR to add 80-bp arms to the *NAT-P_RBT5_* cassette. The 80-bp arms were homologous to the *UME6* 5′ region (Fig. 1A) and were designed to direct homologous integration into the native alleles in the C. albicans genome. The *NAT-*P*_RBT5_* construct was transformed into wild-type and filamentation-defective *efg1Δ/Δ* strains, using a transient CRISPR approach (19), and Nat^R^ transformants were selected and genotyped. Previous studies have shown that the *efg1Δ/Δ* filamentation defect is overcome when *UME6* is overexpressed (20). Therefore, the activity of P*_RBT5_*-*UME6* can be measured by the resulting filamentation level.

**FIG 2:**
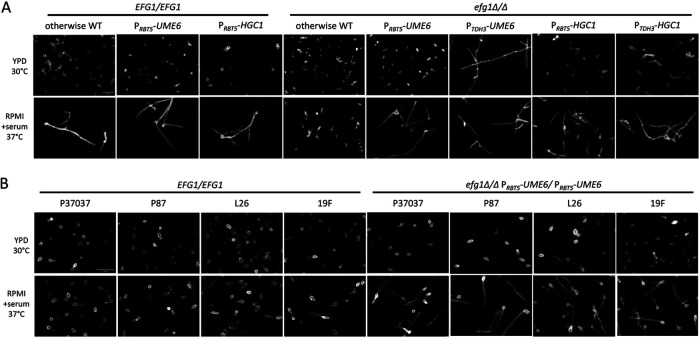
Filamentation assays in YPD and RPMI with serum media. (A) Cells of genotype *EFG1/EFG1* or *efg1Δ/Δ* and otherwise wild type or otherwise homozygous for P*_RBT5_*-*UME6*, P*_TDH3_*-*UME6*, P*_RBT5_*-*HGC1*, or P*_TDH3_*-*HGC1* as indicated were assayed for filamentation under planktonic growth conditions. Strains were grown in YPD medium overnight at 30°C with shaking or transferred to RPMI with 10% serum at 37°C for 4 h after YPD overnight growth with shaking. Fixed cells were stained with calcofluor white for confocal microscopy. White scale bar, 20 μm. (B) *EFG1/EFG1* and *efg1Δ/Δ* P*_RBT5_*-*UME6/*P*_RBT5_*-*UME6* strains from the indicated clinical isolate backgrounds were assayed for filamentation as described for panel A. White scale bar, 20 μm.

We first examined filamentation of the otherwise-wild-type strain homozygous for either *UME6* or P*_RBT5_*-*UME6* (Fig. 2A). We examined a yeast extract-peptone-dextrose (YPD), 30°C overnight culture, which was noninducing for filamentation and iron replete, and an RPMI with serum, 37°C 4-h culture, which was inducing for filamentation and was iron limited. Both strains grew as yeast cells in YPD and formed filaments in RPMI with serum (Fig. 2A). These results matched expectations and indicated that *P_RBT5_*-*UME6* has low activity in YPD at 30°C. A more discerning test of P*_RBT5_*-*UME6* function was provided by responses of *efg1Δ/Δ* mutants homozygous for either *UME6* or P*_RBT5_*-*UME6*. Both strains grew as yeast cells in YPD, but only the P*_RBT5_*-*UME6* strain formed filaments in RPMI with serum (Fig. 2A). Results with the *efg1Δ/Δ UME6/UME6* strain matched expectations, given that Efg1 is required for filamentation. Results with the *efg1Δ/Δ P_RBT5_*-*UME6/*P*_RBT5_*-*UME6* strain indicated that P*_RBT5_*-*UME6* is expressed in RPMI with serum at 37°C. A control *efg1Δ/Δ* P*_TDH3_*-*UME6/*P*_TDH3_*-*UME6* strain, in which *UME6* expression is driven by the constitutive *TDH3* promoter (16), produced hyphae in both YPD and RPMI with serum cultures (Fig. 2A), matching expectations for constitutive *UME6* expression. These results indicate that the transplanted *RBT5* promoter directs regulated expression of *UME6.*

Most studies of gene function have been conducted with the SC5314 type strain of C. albicans and its derivatives. However, the filamentation gene regulatory network is variable among clinical isolates (17). This consideration prompted us to test the *RBT5* promoter in multiple genetic backgrounds. We constructed *efg1Δ/Δ* P*_RBT5_*-*UME6/*P*_RBT5_*-*UME6* strains in the P37037, P87, L26, and 19F backgrounds (21, 22). All strains grew as yeast cells in YPD and formed filaments in RPMI with serum (Fig. 2B). Filamentation levels in RPMI with serum for the *efg1Δ/Δ* P*_RBT5_*-*UME6/*P*_RBT5_*-*UME6* strains were greater than those for the wild-type clinical isolates (Fig. 2B). These results indicate that the transplanted *RBT5* promoter directs expression of *UME6* in multiple strain backgrounds in RPMI with serum medium.

**FIG 3:**
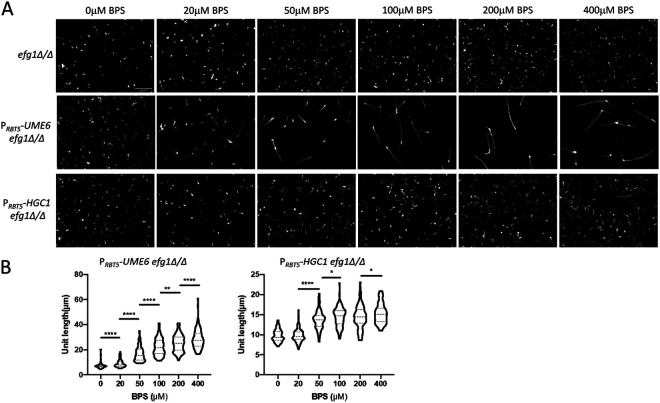
Effect of iron chelation on *RBT5* promoter constructs. (A) Filamentation assays of *efg1Δ/Δ*, *efg1Δ/Δ* P*_RBT5_*-*UME6*/P*_RBT5_*-*UME6*, and *efg1Δ/Δ* P*_RBT5_*-*HGC1*/P*_RBT5_*-*HGC1* strains were grown in YPD plus 0, 20, 50, 100, 200, 300, or 400 μM BPS at 37°C for 4 h with shaking. Fixed cells were stained with calcofluor white for confocal microscopy. The white scale bar (top left panel) represents 50 μm. (B) Cell body lengths were quantified with ImageJ, with a minimum of 100 cells for each BPS concentration. Values shown are means with SD. Pairs of means connected by a horizontal bar are significantly different (Tukey-Kramer test: *, *P* < 0.05; **, *P *< 0.01; ***, *P *< 0.001; ****, *P *< 0.0001).

To establish that iron levels regulate *RBT5* promoter activity in the P*_RBT5_*-*UME6* construct, we tested filamentation of the SC5314-derived *efg1Δ/Δ* P*_RBT5_*-*UME6/*P*_RBT5_*-*UME6* strain under conditions of progressive iron limitation. Iron levels were modulated through chelation with bathophenanthrolinedisulfonic acid (BPS) (23). In YPD medium at 37°C, addition of BPS increased the frequency and length of hyphal filaments (Fig. 3A and B). In an *efg1Δ/Δ* mutant without the P*_RBT5_*-*UME6* construct, BPS did not induce filamentation (Fig. 3A). Therefore, iron limitation increases *RBT5* promoter activity in the P*_RBT5_*-*UME6* allele.

To establish that the *RBT5* promoter can be applied to additional genes, we turned to the *HGC1* gene. *HGC1* encodes a hypha-associated cyclin whose overexpression is sufficient to drive filamentation in a variety of noninducing conditions (24, 25). An *efg1Δ/Δ* P*_RBT5_*-*HGC1/*P*_RBT5_*-*HGC1* strain grew as yeast in YPD culture and produced filaments in RPMI with serum (Fig. 2A). A control *efg1Δ/Δ* P*_TDH3_*-*HGC1/*P*_TDH3_*-*HGC1* strain produced elongated cells in YPD culture and hyphae in RPMI with serum culture (Fig. 2A). Constitutive *HGC1* expression has been reported previously not to override all filament-inducing signals (24), and the results in YPD at 30°C are consistent with those reports. Importantly, P*_RBT5_*-*HGC1* can bypass the dependence of filamentation on Efg1, as expected given that Hgc1 acts downstream of Efg1 (20) and that it is expressed independently of Efg1 from the P*_RBT5_*-*HGC1* allele.

We used BPS titration in YPD at 37°C to confirm that iron levels regulate P*_RBT5_*-*HGC1* activity. Increasing BPS concentrations resulted in increasing filamentation of the *efg1Δ/Δ* P*_RBT5_*-*HGC1/*P*_RBT5_*-*HGC1* strain (Fig. 3A and B). The level of filamentation driven by P*_RBT5_*-*HGC1* was less than that driven by P*_RBT5_*-*UME6* (Fig. 3A and B), a recapitulation of observations with the *TDH3* promoter-driven alleles in YPD (Fig. 2A). These results confirmed that iron limitation increases *RBT5* promoter activity in the P*_RBT5_*-*HGC1* allele.

**FIG 4:**
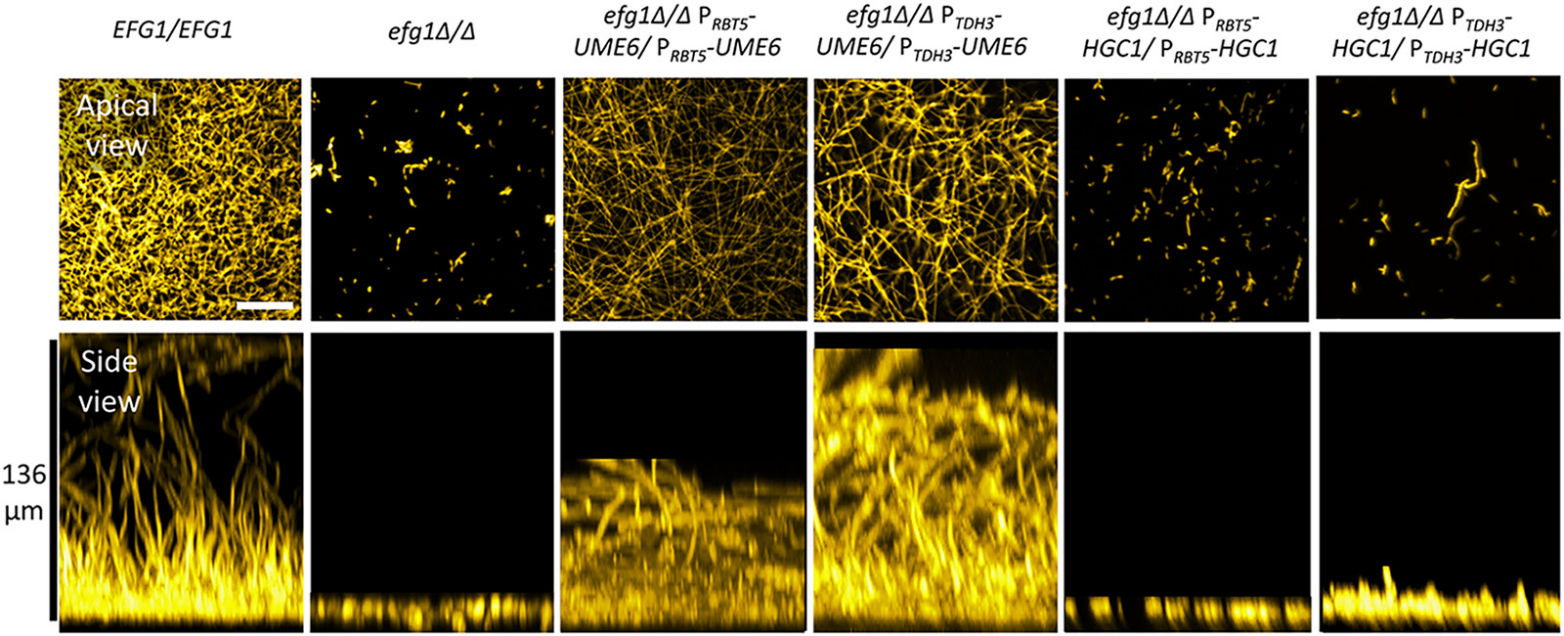
Biofilm assays. Cells of genotype *EFG1/EFG1* or *efg1Δ/Δ* and otherwise wild type or homozygous for P*_RBT5_*-*UME6*, P*_TDH3_*-*UME6*, P*_RBT5_*-*HGC1*, or P*_TDH3_*-*HGC1* as indicated were assayed for biofilm formation after growth in a 96-well plate in RPMI with 10% serum at 37°C for 24 h. Fixed biofilms were stained using calcofluor white, then imaged by confocal microscopy. Representative apical and side views are shown. For apical views, the white scale bar represents 50 μm. For side views, the vertical black scale bar represents 136 μm.

*UME6* and *HGC1* govern biofilm formation as well as hypha formation (20). To assess the utility of *RBT5* promoter activity under biofilm growth conditions, we assayed biofilm formation by wild-type, *efg1Δ/Δ*, *efg1Δ/Δ* P*_RBT5_*-*UME6/*P*_RBT5_*-*UME6* and *efg1Δ/Δ* P*_RBT5_*-*HGC1/*P*_RBT5_*-*HGC1* strains. Biofilms were grown in RPMI with serum at 37°C. The wild-type strain produced biofilm and the *efg1Δ/Δ* strain did not, as expected (Fig. 4). The *efg1Δ/Δ* P*_RBT5_*-*UME6/*P*_RBT5_*-*UME6* strain produced biofilm at levels comparable to an *efg1Δ/Δ* P*_TDH3_*-*UME6/*P*_TDH3_*-*UME6* strain, assayed side-by-side (Fig. 4). The *efg1Δ/Δ* P*_RBT5_*-*HGC1/*P*_RBT5_*-*HGC1* strain failed to produce biofilm, as did a side-by-side *efg1Δ/Δ* P*_TDH3_*-*HGC1/*P*_TDH3_*-*HGC1* strain (Fig. 4). Given that either of two promoter fusions enables *UME6* to bypass the *efg1Δ/Δ* mutation and that neither enables *HGC1* to do so, we infer that the *RBT5* promoter is active under these biofilm growth conditions. The difference in outcomes with P*_RBT5_*-*UME6* and P*_RBT5_*-*HGC1* likely reflects differences in functional activity of Ume6 and Hgc1.

**FIG 5:**
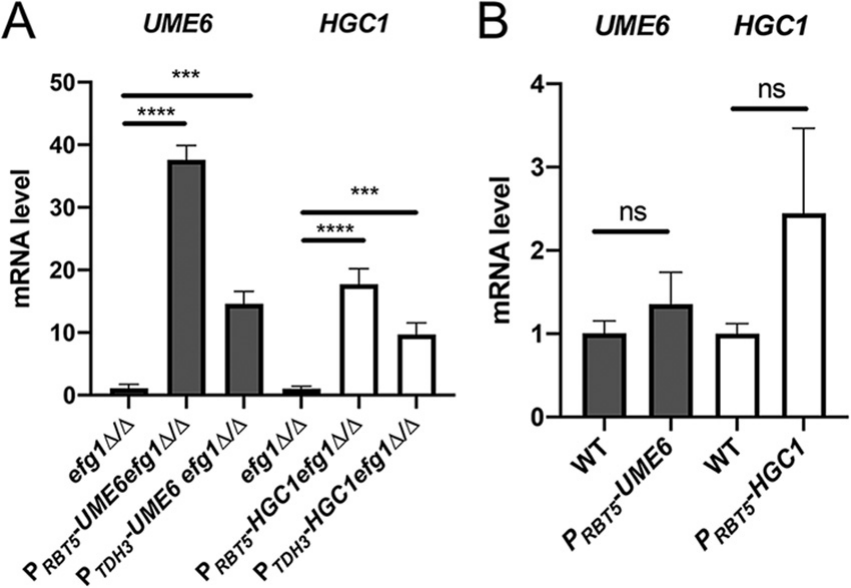
Expression levels of *RBT5* promoter constructs. Strains were grown in RPMI with 10% serum at 37°C for 4 h with shaking. qRT-PCR analysis for RNA levels of *UME6* or *HGC1* under control of their native promoters, the *RBT5* promoter, or the *TDH3* promoter were measured and normalized to the level of *ACT1* mRNA. Results from three independent experiments are shown. Values shown are means (+SD). Pairs of means connected by a horizontal bar are significantly different (Tukey-Kramer test: *, *P* < 0.05; **, *P *< 0.01; ***, *P *< 0.001; ****, *P *< 0.0001). (A) Strains were genotype *efg1Δ/Δ* and homozygous for the hybrid *UME6* or *HGC1* alleles indicated. (B) Strains were genotype *EFG1/EFG1* and homozygous for the hybrid *UME6* or *HGC1* alleles indicated.

To test expression levels driven by the *RBT5* promoter, we conducted reverse transcription-quantitative PCR (RT-qPCR) assays with cells grown in RPMI with serum medium. RNA levels of P*_RBT5_*-*UME6* or P*_RBT5_*-*HGC1* alleles were 20- to 40-fold higher than for the native alleles in the parent *efg1Δ/Δ* strain (Fig. 5A). RNA levels were similar in magnitude for alleles driven by the *RBT5* and *TDH3* promoters (Fig. 5A). In a wild-type background, RNA levels driven by the *RBT5* promoter and the native *UME6* or *HGC1* promoter were comparable (Fig. 5B). These results confirmed that the *RBT5* promoter can drive high-level expression of C. albicans genes.

### *RBT5* promoter activity during infection.

**FIG 6:**
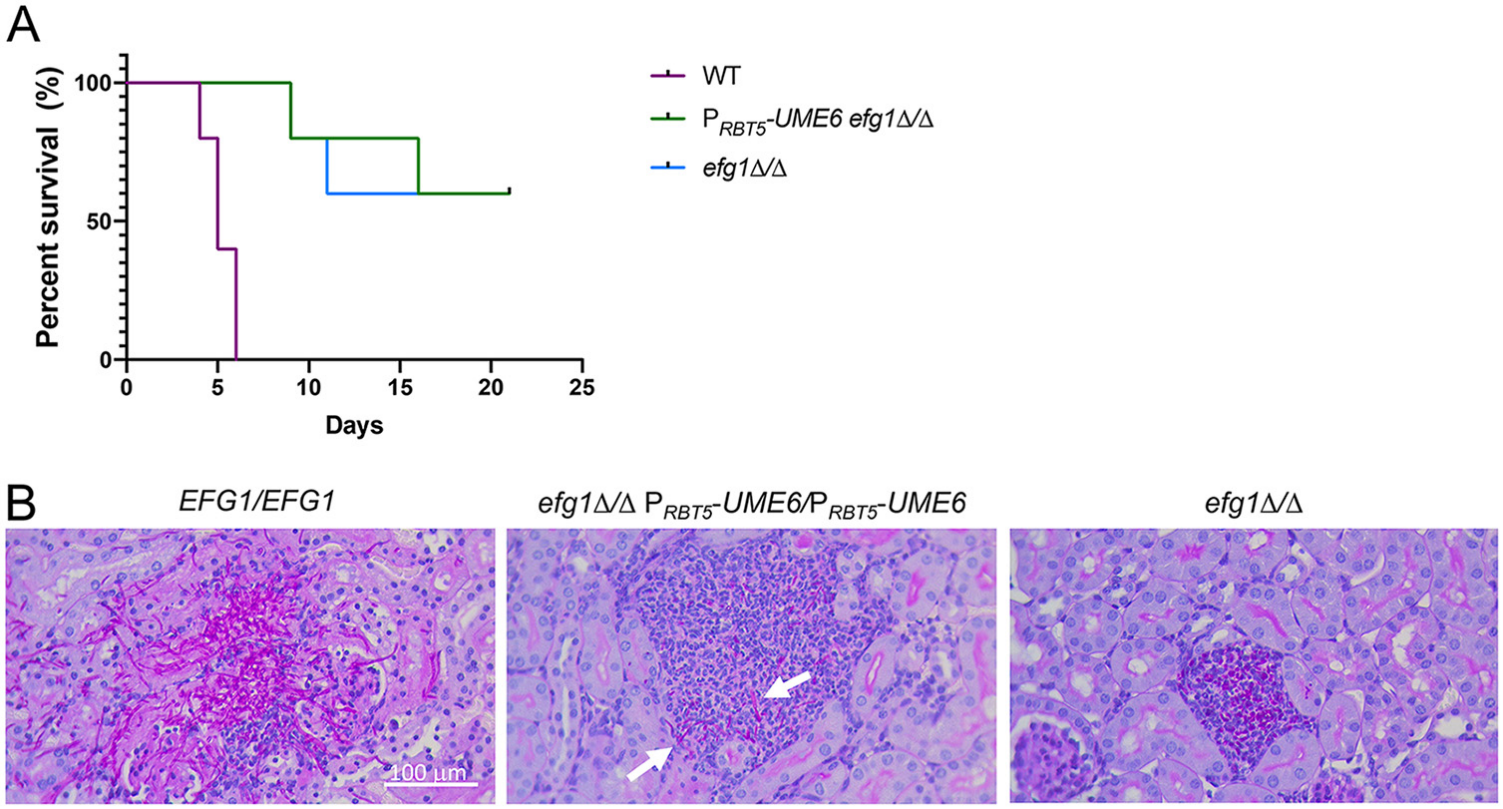
Virulence tests. (A) Survival of mice infected with the indicated strains. (B) Histology sections of infected mouse kidneys at 2 days postinfection with the strains indicated. White arrows point to filamentous cells produced by the *efg1Δ/Δ* P*_RBT5_*-*UME6/*P*_RBT5_*-*UME6* strain. White scale bar, 100 μm.

To determine whether the *RBT5* promoter can drive gene expression during infection, we used a mouse model of hematogenously disseminated infection (26). We compared mouse survival after infection with wild-type, *efg1Δ/Δ*, and *efg1Δ/Δ* P*_RBT5_*-*UME6/*P*_RBT5_*-*UME6* strains. Based on host survival times, the wild-type strain was virulent and the *efg1Δ/Δ* mutant was attenuated (Fig. 6A), as expected from previous studies (27). The *efg1Δ/Δ* P*_RBT5_*-*UME6/*P*_RBT5_*-*UME6* strain was no more virulent than the *efg1Δ/Δ* strain (Fig. 6A). To determine whether the P*_RBT5_*-*UME6* allele may drive filamentation during infection, we conducted histopathological examination of kidney sections from mice at 2 days postinfection. The wild-type strain produced abundant filaments in the kidney, while the *efg1Δ/Δ* mutant produced none (Fig. 6B). The *efg1Δ/Δ* P*_RBT5_*-*UME6/*P*_RBT5_*-*UME6* strain produced short filaments (Fig. 6B). The ability of the P*_RBT5_*-*UME6* allele to modify the *efg1Δ/Δ* mutant phenotype indicates that the P*_RBT5_*-*UME6* allele is expressed and functional during infection. It will be interesting in future detailed studies to define the spectrum of genes that are expressed by the *efg1Δ/Δ* P*_RBT5_*-*UME6/*P*_RBT5_*-*UME6* strain during infection. Prior *in vitro* studies indicated that *UME6* overexpression in an *efg1Δ/Δ* mutant is not sufficient to activate many known virulence-related genes, including *ECE1*, *ALS3*, and *HWP1* (20). The *efg1Δ/Δ* P*_RBT5_*-*UME6/*P*_RBT5_*-*UME6* strain may be a useful platform to define Efg1-dependent genes that promote virulence.

## MATERIALS AND METHODS

### Strains and culture conditions.

All strains are listed in Table 1. C. albicans strains SC5314, P37037, P87, L26, and 19F (21, 22) and their derived *efg1Δ/Δ* mutants were used as transformation recipients. Construction of the *efg1Δ/Δ* mutants in clinical isolates has been described previously (17) and will be detailed for strains P37037, L26, and 19F separately. Fungal strains were grown at 30°C in YPD (2% Bacto peptone, 2% dextrose, 1% yeast extract) with shaking. For phenotypic assays and biofilm assays, strains were grown in liquid RPMI 1640 medium (Sigma-Aldrich, St. Louis, MO) adjusted to pH 7.4 and supplemented with 10% fetal bovine serum (Atlanta Biologicals, Inc., Flowery Branch, GA). C. albicans transformants were selected on YPD plus NAT (2% Bacto peptone, 2% dextrose, 1% yeast extract and 400 μg/mL [Werner BioAgents]) for nourseothricin-resistant (Nat^R^) isolates. All strains were stored as glycerol stocks at −80°C.

**TABLE 1:** Strains

Strain no.	Strain	Phenotype	Genotype
YM1	SC5314	His^+^ Nat^S^	SC5314 wild type
MC84	SC5314 *efg1*Δ/Δ	His^+^ Nat^S^	*efg1*Δ::*r1HIS1r1/efg1*Δ::*r1HIS1r1 his1*Δ::*r3/his1*Δ::*r3*
MC431	SC5314 *efg1*Δ/Δ P*_TDH3_-UME6*	His^+^ Nat^R^	*efg1*Δ::*r1HIS1r1/efg1*Δ::*r1HIS1r1 UME6*::P*_TDH3_-UME6/UME6::P_TDH3_-UME6 his1*Δ::*r3/his1*Δ::*r3*
MC153	P37037 *efg1*Δ/Δ	His^+^ Nat^S^	*efg1*Δ::*r1HIS1r1/efg1*Δ::*r1HIS1r1 his1*Δ::*r3/his1*Δ::*r3*
MC144	P87 *efg1*Δ/Δ	His^+^ Nat^S^	*efg1*Δ::*r1HIS1r1/efg1*Δ::*r1HIS1r1 his1*Δ::*r3/his1*Δ::*r3*
MC147	L26 *efg1*Δ/Δ	His^+^ Nat^S^	*efg1*Δ::*r1HIS1r1/efg1*Δ::*r1HIS1r1 his1*Δ::*r3/his1*Δ::*r3*
MC150	19F *efg1*Δ/Δ	His^+^ Nat^S^	*efg1*Δ::*r1HIS1r1/efg1*Δ::*r1HIS1r1 his1*Δ::*r3/his1*Δ::*r3*
YM150	SC5314 *efg1*Δ/Δ P*_RBT5_-UME6*	His^+^ Nat^R^	*efg1*Δ::*r1HIS1r1/efg1*Δ::*r1HIS1r1 UME6*::P*_RBT5_-UME6/UME6::P_RBT5_-UME6 his1*Δ::*r3/his1*Δ::*r3*
YM154	SC5314 P*_RBT5_-UME6*	His^+^ Nat^R^	*UME6*::P*_RBT5_-UME6/UME6*::P*_RBT5_-UME6*
YM155	P37037 *efg1*Δ/Δ P*_RBT5_-UME6*	His^+^ Nat^R^	*efg1*Δ::*r1HIS1r1/efg1*Δ::*r1HIS1r1 UME6*::P*_RBT5_-UME6/UME6::P_RBT5_-UME6 his1*Δ::*r3/his1*Δ::*r3*
YM156	P87 *efg1*Δ/Δ P*_RBT5_-UME6*	His^+^ Nat^R^	*efg1*Δ::*r1HIS1r1/efg1*Δ::*r1HIS1r1 UME6*::P*_RBT5_-UME6/UME6::P_RBT5_-UME6 his1*Δ::*r3/his1*Δ::*r3*
YM157	L26 *efg1*Δ/Δ P*_RBT5_-UME6*	His^+^ Nat^R^	*efg1Δ::r1HIS1r1/efg1Δ::r1HIS1r1, UME6::P_RBT5_-UME6/UME6::P_RBT5_-UME6 his1Δ::r3/his1Δ::r3*
YM158	19F *efg1*Δ/Δ P*_RBT5_-UME6*	His^+^ Nat^R^	*efg1*Δ::*r1HIS1r1/efg1*Δ::*r1HIS1r1 UME6*::P*_RBT5_-UME6/UME6::P_RBT5_-UME6 his1*Δ::*r3/his1*Δ::*r3*
YM160	SC5314 P*_RBT5_-HGC1*	His^+^ Nat^R^	*HGC1*::P*_RBT5_-HGC1/HGC1*::P*_RBT5_-HGC1*
YM161	SC5314 *efg1*Δ/Δ P*_RBT5_-HGC1*	His^+^ Nat^R^	*efg1*Δ::*r1HIS1r1/efg1*Δ::*r1HIS1r1 HGC1*::P*_RBT5_-HGC1/HGC1::P_RBT5_-HGC1 his1*Δ::*r3/his1*Δ::*r3*
ASM350	SC5314 *efg1*Δ/Δ P*_TDH3_-HGC1*	His^+^ Nat^R^	*efg1*Δ::*r1HIS1r1/efg1*Δ::*r1HIS1r1 HGC1*::P*_TDH3_-HGC1/HGC1::P_TDH3_-HGC1 his1*Δ::*r3/his1*Δ::*r3*

### Plasmids and DNA.

Primers are listed in Table 2 and plasmids are listed in Table 3.

**TABLE 2:** Primers

Primer name	Sequence^*a*^
RBT5/F	cgattcgatactaacgcaatcactatttttaatgatcctacttctatcccgaaaaag
RBT5/R	aacgcgttgggagctctcccatatggtagtattagttattagtgatagttagtgaagaattag
OE backbone/F	atatgggagagctcccaac
OE backbone/R	tagtgattgcgttagtatcgaatc
Multipromoter UME6/F	aaaaaagaattcttcgtaatatctatatagatatcttcatttaattttcttggttgtttgatattactttgttgtacttt**ATCAAGCTTGCCTCGTCCCC**
Multipromoter HGC1/F	cccaaactatacttcccaataaaagatagaaactcgcttacaacaacacaatcctgaagattattaaatctctaattttc**ATCAAGCTTGCCTCGTCCCC**
Multipromoter UME6/R	ttcaactttattgtatcttctccataaggcgaatttggtgctgaagaagttgaatcgggtgtaaccatatgggtaatcat**CGTTGGGAGCTCTCCCAATG**
Multipromoter HGC1/R	atgtttttgtatggatgttgttgttgttgtttttgttgtgaaattgattttggagttaatggtttagttatatttatcat**CGTTGGGAGCTCTCCCAATG**
CaCas9/for	Atctcattagatttggaacttgtgggtt
CaCas9/rev	Ttcgagcgtcccaaaaccttct
UME6p sgRNA/F	caagaaaattatcaaattctgttttagagctagaaatagcaagttaaa
UME6p SNR52/R	agaatttgataattttcttgcaaattaaaaatagtttacgcaagtc
HGC1p sgRNA/F	gtgtgtatagtgtagtatccgtgttttagagctagaaatagcaagttaaa
HGC1p SNR52/R	acggatactacactatacacaccaaattaaaaatagtttacgcaagtc
UME6 qRT/F	tccactttaccattatccaagtctactc
UME6 qRT/R	gtgttgataatgaatgaactaaatttgccc
HGC1 qRT/F	caccaccacaaatgcattctca
HGC1 qRT/R	atgaggtgcaggaagctgac
UME6p OE NAT1 TDH3/F	gggaaaaaagaattcttcgtaatatctatatagatatcttcatttaattttcttggttgtttgatattactttgttgtac**ATCAAGCTTGCCTCGTCCCC**
UME6p OE TDH3 NAT1/R	ttcaactttattgtatcttctccataaggcgaatttggtgctgaagaagttgaatcgggtgtaaccatatgggtaatcat**TGTTAATTAATTTGATTGTAAAGTTTGTTGATG**
UME6p check up/F	gagagttttaatcaattagaaaccaacagagg
UME6p check int/R	cgaatgacaaagttaagtcaaaaattggacc
NAT1 check/R	tcaatggtggatcaactggaacttc
HGC1 OE/F	cccaaactatacttcccaataaaagatagaaactcgcttacaacaacacaatcctgaagattattaaatctctaattttc**ATCAAGCTTGCCTCGTCCCC**
HGC1 OE/R	atgtttttgtatggatgttgttgttgttgtttttgttgtgaaattgattttggagttaatggtttagttatatttatcat**TGTTAATTAATTTGATTGTAAAGTTTGTTGATG**
HGC1 check/F	cttacattttagacgaccaacggatactaca
HGC1 check/R	cttcgattgaaggatcatttaaagaccattctaaa

a Nucleotides in bold uppercase letters are complementary to the pTH10 plasmid template.

**TABLE 3:** Plasmids

Plasmid name	Description	Marker	Reference
pNAT	NAT1 marker	ampR	19
pCJN542	NAT1-TDH3 promoter	ampR	16
pV1093	CaCas9/sgRNA expression vector	ampR	28
pMH01	pRS424 carrying C.d.HIS1 from pSN52 at KpnI site	ampR	30
pMH02	pRS424 carrying C.d.HIS1 from pSN52 at SapI site	ampR	30
pTH10	RBT5 promoter cassette	ampR	This study

### Generation of plasmid pTH10.

To construct plasmid pTH10, we modified plasmid pCJN542 (16) by replacing the *TDH3* promoter with the *RBT5* promoter. The 1-kb *RBT5* promoter was amplified by PCR from SC5314 genomic DNA using primers RBT5/F and RBT5/R. The two primers were flanked by a 25-bp segment of homology upstream or downstream of the *TDH3* promoter in order to make ligation with the other parts of plasmid. The 4.2-kb backbone fragment containing the ORI sequence, Amp^R^, and Nat^R^ was amplified by PCR from pCJN542 using primers OE backbone/F and OE backbone/R. PCR products of the *RBT5* promoter and backbone were purified and ligated by using a NEBuilder HiFi DNA assembly cloning kit (NEB, USA) to yield plasmid pTH10.

### P*_RBT5_* cassettes.

The P*_RBT5_* cassette for *UME6* overexpression containing Nat^R^ and the *RBT5* promoter was amplified by PCR using primers multipromoter UME6/F and multipromoter UME6/R, containing 80 bp of homology upstream or downstream of the *UME6* promoter region. The P*_RBT5_* cassette for *HGC1* overexpression containing Nat^R^ and the *RBT5* promoter was amplified by PCR using primers multipromoter HGC1/F and multipromoter HGC1/R, containing 80 bp of homology upstream or downstream of the *HGC1* promoter region.

### P*_TDH3_* cassettes.

The *P_TDH3_* cassette for *UME6* overexpression containing Nat^R^ and the *TDH3* promoter was amplified from plasmid pCJN542 by PCR using primers UME6p OE NAT1 TDH3/F and UME6p OE TDH3 NAT1/R, containing 80 bp of homology upstream or downstream of the *UME6* promoter region. The P*_TDH3_* cassette for *HGC1* overexpression containing Nat^R^ and the *TDH3* promoter was amplified from plasmid pCJN542 by PCR using primers multi HGC1 OE/F and HGC1 OE/R, containing 80 bp of homology upstream or downstream of the *HGC1* promoter region.

### Other DNA cassettes.

The approximately 5-kb CaCas9 cassette containing an *ENO1* promoter, the CaCas9 open reading frame (ORF), and a *CYC1* terminator was amplified from pV1093 (28) using primers CaCas9/for and CaCas9/rev. The single guide RNA (sgRNA) cassettes for the *UME6* or *HGC1* 5′ region, containing the SNR52 promoter, guide sequence, and sgRNA scaffold sequence, were amplified via split-joint PCR as previously described (19) using primer pairs UME6p-sgRNA/F and UME6p-sgRNA/R and HGC1p1-sgRNA/F and HGC1p-sgRNA/R, respectively. PCR products were purified and concentrated with the GeneJET PCR purification kit (Thermo Fisher Scientific, Inc.).

### C. albicans transformation.

Transformation was done via the transient CRISPR system (19). The P*_RBT5_* cassette (2.2 μg) was cotransformed with the CaCas9 cassette (1.5 μg) and sgRNA cassette (1.5 μg), using the lithium acetate transformation method (28). Nat^R^ transformants were selected and genotyped. The *UME6* overexpression cassette was verified by PCR from genomic DNA using primers UME6p check up/F and UME6p check int/R for absence of the *UME6* promoter and primers UME6p check up/F and NAT1 Check/R for presence of the *NAT1* marker. The *HGC1* overexpression cassette was verified by PCR from genomic DNA using primers HGC1 check/F and HGC1 check/R for absence of the *HGC1* promoter and using primers HGC1 check/F and NAT1 Check/R for presence of the *NAT1* marker.

### Filamentation assay.

To assay hyphal formation, strains were inoculated from YPD overnight cultures to an OD_600_ of 0.4 into 5 mL of RPMI with 10% serum in glass test tubes. Cells were grown for 4 h at 37°C with shaking, then collected by centrifugation and fixed with 4% formaldehyde for 15 min. Fixed cells were washed in phosphate-buffered saline (PBS), stained with 200 ng/μL calcofluor white, and imaged using a slit-scan confocal optical unit on a Zeiss Axiovert 200 microscope with a Zeiss C-Apochromat 40×, 1.2 numerical aperture water immersion objective. Lengths of hyphal units, i.e., the distance between septa on hyphae, were quantified using ImageJ. At least 100 interseptal distance measurements were taken from 3 separate views.

### Biofilm assay.

Biofilm formation was assayed in 96-well plates (Greiner 96 wells, catalog number 655090). Cells were transferred to 100 μL of prewarmed RPMI with 10% serum to an OD_600_ of 0.3 from YPD overnight cultures. Cells were incubated at 37°C for 90 min with mild shaking (60 rpm) to allow for adherence to the bottom of 96-well plates, and then each well was gently washed twice with PBS to remove nonadhered cells. Next, cells were incubated in 100 μL of prewarmed RPMI with 10% serum at 37°C for 24 h with mild shaking. Then, the medium was removed and biofilms were fixed by incubation with 100 μL of 4% formaldehyde in PBS solution for 1 h and then gently washed twice with PBS. Biofilms were stained with 200 μg/mL calcofluor white overnight at room temperature with mild shaking, and then each well was gently washed twice with PBS. Finally, we used 100% thiodiethanol (TDE) followed by 50% TDE in PBS to clarify biofilms. Biofilms were imaged using a Zeiss LSM 710 inverted confocal microscope and analyzed with Fiji ImageJ.

### RNA extraction and qPCR.

RNA extractions were conducted as previously described (29). Briefly, strains were inoculated in triplicate from overnight cultures cells into 25 mL of RPMI with 10% serum at 37°C with shaking for 4 h to an initial OD_600_ of 0.2. Cells were harvested by vacuum with filtration. Then, cells were lysed using a BeadBeater and a Qiagen RNeasy minikit (catalog number 74104). RNA was isolated with the RNeasy kit and reverse transcribed to cDNA using the iScript gDNA clear cDNA synthesis kit (catalog number 172-5034). Then, qPCR was performed using iQ SYBR green supermix (catalog number 170-8880). *UME6* and *HGC1* expression levels were normalized to the *ACT1* gene and compared using the threshold cycle ΔΔ*C_T_* method. Differences between strains were analyzed with the Tukey-Kramer test.

### Animal studies.

Virulence of C. albicans strains was determined using the mouse model of hematogenously disseminated candidiasis (HDC) in 5- to 6-week-old male BALB/c mice (Taconics). Mice were injected via the lateral tail vein with 5 × 10^5^ yeast and monitored for survival over a 3-week period. For histology, two additional mice were infected via the tail vein with 1 × 10^6^ yeast and sacrificed after 2 days, after which a kidney from each mouse was fixed in zinc-buffered formalin and embedded in paraffin. Thin sections were cut and then stained with Periodic acid-Schiff and imaged by light microscopy. Animal work was approved by the Lundquist Institute for Biomedical Research at Harbor-UCLA Medical Center and carried out in accordance with the National Institutes of Health guidelines for the ethical treatment of animals.

### Data availability.

Plasmid pTH10 and its sequence have been deposited with Addgene.
